# Co-Association Matrix-Based Multi-Layer Fusion for Community Detection in Attributed Networks

**DOI:** 10.3390/e21010095

**Published:** 2019-01-20

**Authors:** Sheng Luo, Zhifei Zhang, Yuanjian Zhang, Shuwen Ma

**Affiliations:** 1 Department of Computer Science and Technology, Tongji University, Shanghai 201804, China; 2 State Key Laboratory for Novel Software Technology, Nanjing University, Nanjing 210023, China; 3 Research Center of Big Data and Network Security, Tongji University, Shanghai 200092, China

**Keywords:** community detection, attributed graph, complex networks, information fusion, data inconsistency

## Abstract

Community detection is a challenging task in attributed networks, due to the data inconsistency between network topological structure and node attributes. The problem of how to effectively and robustly fuse multi-source heterogeneous data plays an important role in community detection algorithms. Although some algorithms taking both topological structure and node attributes into account have been proposed in recent years, the fusion strategy is simple and usually adopts the linear combination method. As a consequence of this, the detected community structure is vulnerable to small variations of the input data. In order to overcome this challenge, we develop a novel two-layer representation to capture the latent knowledge from both topological structure and node attributes in attributed networks. Then, we propose a weighted co-association matrix-based fusion algorithm (WCMFA) to detect the inherent community structure in attributed networks by using multi-layer fusion strategies. It extends the community detection method from a single-view to a multi-view style, which is consistent with the thinking model of human beings. Experiments show that our method is superior to the state-of-the-art community detection algorithms for attributed networks.

## 1. Introduction

A large number of complex systems in the real world are often represented as complex networks, such as communication systems, biological systems, social systems, traffic systems and World Wide Web (WWW), etc. [1,2]. One of the most important characteristics of complex networks is community structure [3]. Detecting community structure is one of the fundamental problems in complex network analysis. By detecting the community structure, one could easily understand not only the intrinsic characteristics of complex networks, but also its evolutionary trends. The community detection problem has become one of the hot spots in the field of complex network analysis. Many experts and scholars have proposed a large number of excellent algorithms to find the community structure. Early work [4,5,6,7,8,9,10] of community detection is mainly focused on networks without node and edge attributes. Most community detection algorithms for complex networks are proposed mainly based on graph partitioning [4], spectral methods [11], and graph cut [5]. In addition, some methods [12,13] use entropy theory to measure the uncertainty in complex networks and improve the performance of algorithms such as community detection. Although these methods would work well, they still do not generate well partitioned and easily-understood community structure in a comprehensive view for complex networks because of the lack of attribute information.

In general, attribute information describes the inherent characteristics of each node and edge and influences the community structure. As the attribute information of node and edge becomes more and more abundant, there are many studies that are beginning to take attribute information into account in the community detection algorithm [14,15,16,17,18,19]. However, information fusion is a big challenge [20,21] existing in most community detection algorithms [22], due to the complexity of multi-source heterogeneous data. In attributed networks, how to fuse the topological structure and attribute information of complex networks is an important factor to reasonably recognize and understand community structure. A natural strategy is to calculate the similarity matrix from network structure and node attribute data, respectively, and combine these similarities into a mixture form as the input of community detection algorithms requiring similarity matrix [23,24]. For example, Neville et al. [23] use the matching coefficient similarity metric $sim^{a}\left(o_{i},o_{j}\right)$ to calculate the similarity for node pair $\left(o_{i},o_{j}\right)$ by using node attributes, i.e.,

(1) $$sim^{a}\left(o_{i},o_{j}\right)=\sum_{f}\delta \left(f\left(o_{i}\right),f\left(o_{j}\right)\right),$$

where *f* is an attribute (or feature), $o_{i}$ is a node in an attributed network, $f(o_{i})$ is the value of $o_{i}$ in attribute *f*, δ(b,d) is a delta function, that is, δ(b,d)=1 if b=d; otherwise, it is 0. Then, the following strategy is used to fuse the attribute similarity and topological similarity,

(2) $$sim^{m}\left(o_{i},o_{j}\right)=\left\{\begin{array}{cc} sim^{a}\left(o_{i},o_{j}\right), & if\;(o_{i},o_{j})\;\mathrm{is}\;\mathrm{an}\;\mathrm{edge}\;\mathrm{in}\;\mathrm{an}\;\mathrm{attributed}\;\mathrm{network},\\ 0, & \mathrm{otherwise}. \end{array}\right.$$

Obviously, it is suitable only for categorical attributes. Steinhaeuser et al. [24] extends it to deal with both categorical and continuous attributes. The drawback of this sort of methods is that the fusion strategy will induce loss of information when there is no edge between node pairs. Therefore, another fusion strategy is proposed which employees linear combination methods to calculate mixture similarity considering both network topological structure and node attribute data [19,25]. For example, Combe et al. [19] provides a mixture distance between nodes as follows:

(3) $$dist^{m}\left(o_{i},o_{j}\right)=\alpha \cdot dist^{a}\left(o_{i},o_{j}\right)+\left(1-\alpha \right)\cdot dist^{s}\left(o_{i},o_{j}\right),$$

where $dist^{a}\left(o_{i},o_{j}\right)$ and $dist^{s}\left(o_{i},o_{j}\right)$ are the attribute and topological structure similarity, respectively, between nodes $o_{i}$ and $o_{j}$ and 0≤α≤1 is a weighting parameter to adjust which part of similarity is more important. Once the similarity is obtained, it will be used as the input parameter for similarity-based community detection algorithms. Without loss of generality, suppose that the parameter α is a random variable and the truth distribution of α is p(α); then, it would induce the uncertainty, H(p,q), between *p* and *q*, where *q* is the selected parameter distribution of α in the algorithm, i.e.,

(4) $$H\left(p,q\right)=-\int_{\alpha }p\left(\alpha \right)\log q\left(\alpha \right).$$

In fact, if the distribution *q* of α is not able to approximate *p*, then the uncertainty of the system will increase, that is, the cross entropy H(p,q) will become larger, which affects the stability and accuracy of the community detection algorithms. Although these methods could work well in the attributed graph, they still remain some problems such as loss of information, uncertainties for weighting parameter, results depending on expert experience, etc.; in particular, the fusion strategy is a big challenge to mix the multi-source heterogeneous data. We summarize these methods as the community detection algorithm fusing at a lower-layer.

In response to these challenges, in this paper, we propose a two-layer representation for attributed networks and a community detection algorithm with multi-layer fusion strategy considering both network topological structure and node attribute data. Unlike the fusion strategy directly combining the similarity from multi-source data at lower-layer, we first generate the community structure from the raw data, i.e., network topological data and node attributes, respectively. After the first step, we would obtain a bag of community partitions, which are called the higher-layer representation of raw data. As is known to all, there exists data inconsistency in the raw data (lower-layer), and it would induce uncertainties to partition community structure. In addition, slight variations in the lower-layer will result in a large influence for detected community structures. If we fuse multi-source data with the two-layer representation, then it would help to reduce data inconsistency and generate optimal community partitions which are less sensitive to the variations at the lower-layer representation. Therefore, we proposed a weighted co-association matrix-based fusion algorithm (WCMFA) to generate optimum community structure from the two-layer representation of raw data, by using ensemble learning technology. The experiments proved that our method is effective and outperforms state-of-the-art community detection algorithms for attributed networks.

The contributions of the paper are summarized as follows:

- We propose a two-layer representation for attributed networks. In the view of the representation, the lower-layer representation is the raw data from network topological structure and node attributes, respectively, while the higher-layer representation is a set of community partitions that are generated from lower-layer data by using the existing community detection algorithms.
- We propose a weighted co-association matrix-based community ensemble method for community detection in attributed networks. In order to reduce the uncertainty and data inconsistency, the WCMFA employs the co-association matrix to learn optimum community structure with the two-layer representation of raw data.
- We also empirically evaluate the effectiveness of WCMFA. The experiment results show that our proposed community detection algorithm, WCMFA, is the optimal choice to detect community structure in attributed networks.

The rest of the paper is organized as follows. In Section 2, we introduce some related work from the viewpoint of networks with attributes and networks without attributes. In Section 3, we first give a glimpse of our proposed method. Then, we develop the two-layer representation of attributed networks and define the weighted co-association matrix. Finally, we design the community detection algorithm, WCMFA, with a two-layer fusion strategy. In Section 4, we evaluate the performance of WCMFA compared with the state-of-the-art community detection algorithms for attributed networks. In Section 5, we draw a conclusion of this work.

## 2. Related Work

According to the attributes of network nodes and edges, complex networks can be classified into two categories as attributed networks and non-attributed networks.

*Related work of community detection on networks without attribute information.* The Kernighan–Lin algorithm [4] is one of the community detection methods which employs a greedy strategy to partition networks by introducing a gain function to evaluate the quality of communities, based on graph theory. Earl [11] proposed a method for partitioning networks into a given number of subsets that the number of edges connecting the various subsets is a minimum, based on the spectral characteristic of a Laplacian matrix. Spectral partition methods are expensive, since they require the computation of the eigenvector corresponding to the second smallest eigenvalue. Therefore, researchers proposed some community detection algorithms by using multilevel graph partitioning strategy [26], which reduces the size of the graph by collapsing vertices and edges, partitions the smaller graph, and then uncoarsens it to construct a partition for the original graph. Although the community detection algorithm based on graph partition can work well, the drawback of its is obvious. That is, the size of the two subgraphs must be specified first; otherwise, the correct result will not be obtained. For cases where the number of communities is unknown, Girvan and Newman [5,6] proposed a community detection algorithm, named GN, which obtains community structure by continuously deleting the edges of the network with high betweenness value until there are no edges between the communities. This kind of algorithm does not need to know more additional information; only the topology of the network is required. However, the time complexity of this kind of algorithms is $O(n^{3})$, where *n* is the number of nodes in a network. It is not easy to stop the algorithm, due to the terminate condition not being clear. Modularity [27] is one of the terminate conditions that is used to evaluate the quality of community partitioning. Some community detection algorithms with the modularity optimum strategy are proposed [8,28,29,30,31]. There also exists some modularity maximization strategy embedded community detection algorithms such as spectral methods [32,33], sampling technique [34], greedy algorithms [8,35] and mathematical programming [36], etc. However, more and more research [37,38,39] papers find that the modularity optimum strategy based community detection algorithms have some limitations, such as the resolution limit [40] and extreme degeneracy [41]. Moreover, it was not possible to discover small communities in networks with varying community size [42], and there were expensive computation costs. However, in terms of computational complexity, the label propagation algorithm (LPA) [7] is a simple and time-efficient method for community detection. In LPA-based methods [7,9,10], every node is initialized with a unique label and, at each step, every node updates its label according to the labels that its neighbors have. However, the LPA-based methods often suffer unstable calculation results, that is, the detected community structure is unstable. In addition, there also exists a category of community detection algorithms that use clustering techniques such as graph-partitioning-based clustering [43], spectral-analysis-based clustering [33], hierarchical-based clustering [44] and density-based clustering [45], etc.

*Related work of community detection on attributed networks.* The attributed network (or attributed graph) [15,46] is a kind of important complex network, which has both topological structures and node attributes. In the attributed network context, the topological structure represents the interactions between nodes and the attributes describe the inherent characteristics of each node in the network. However, most community detection algorithms do not apply directly to attributed networks well, due to the lack of attribute information of nodes or edges. Neville et al. [23] proposed a clustering framework to detect community structure, by using a similarity metric that combines structure and attribute information. The similarity metric first employs the matching coefficient similarity metric to quantify the attribute similarity for every two nodes. Then, it is multiplied by 1 as the mixture similarity, if there exists an edge between nodes; otherwise, it is 0. Some community detection algorithms with mixture similarities that combine structure and attribute similarity by multiplying them together are proposed for attributed networks such as [18,24]. Unlike the methods storing their attribute information inside the edges of the network, some researchers [19,25] treat the structural similarity and the attribute similarity as two different similarities and provide a linear combination strategy to mix these similarities as the input of distance-based clustering methods to discover community structure. In addition to community detection algorithms based on distance-based clustering, some methods are random-walk-based approaches [47,48], statistical-inference-based algorithms [16,17,49,50] and subspace-clustering-based approaches [51,52]. However, these methods still use the lower-layer fusion strategy.

## 3. Proposed Method

In this section, we present our weighted co-association matrix-based fusion algorithm for community detection. We first introduce the necessary notation and framework of our method. Then, we describe the details of the method.

### 3.1. Notation and Method Overview

Let G=(V,E,A) be an attributed network, and let A,G=(V,E) be the node attribute set and the network topological structure of the attributed network, respectively. In general, a network (a.k.a graph) G=(V,E) consists of a node set V={u|u∈1…N} and an edge set E={〈u,v〉|∀u,v∈V}. The node attribute set A describes the feature set of each node $v\in R^{d}$ in node set *V*. The goal of the community detection is to learn an optimum node partition according to the feature set of attributed network considering both network topological structure and node attributes data.

**Figure 1:**
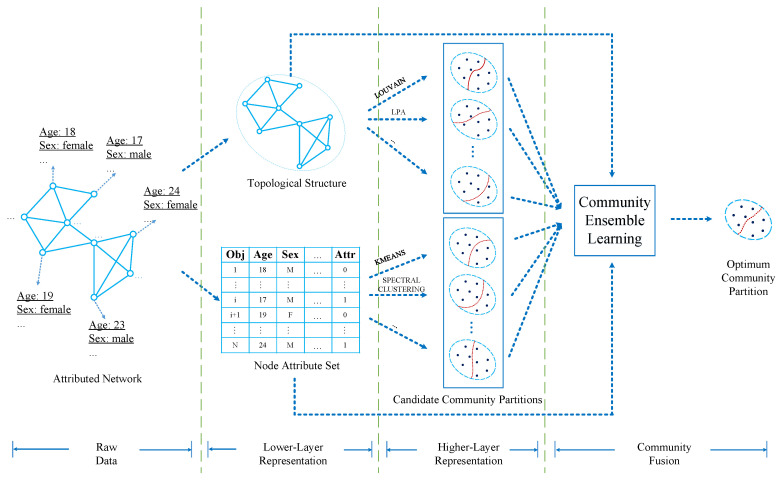
The framework of weighted co-association matrix-based fusion algorithm (WCMFA).

In order to implement the goal, we propose a novel community detection method, i.e., WCMFA. Furthermore, the framework of WCMFA is shown in Figure 1. In the framework, we first divide attributed network data into two categories, i.e., topological structure G(V,E) and node attribute set A. Then, it applies community detection algorithms and clustering algorithms to G(V,E) and A, respectively. In addition, it will generate a set of candidate community partitions from each branch.

Consider *N* candidate community partitions P from the data G(V,E) and A. The P is defined as

(5) $$P=\{P^{1},P^{2},\ldots ,P^{N}\}$$

and

(6) $$\begin{array}{cc} P^{1} & =\{C_{1}^{1},C_{2}^{1},\ldots ,C_{k_{1}}^{1}\},\\ \vdots & \\ P^{N} & =\{C_{1}^{N},C_{2}^{N},\ldots ,C_{k_{N}}^{N}\}, \end{array}$$

where $C_{j}^{i}$ is the *j*th community in the community partition $P^{i}$, which has $k_{i}$ communities and $n_{j}^{i}$ is the cardinality of $C_{j}^{i}$, with $\sum_{j=1}^{k_{i}}n_{j}^{i}=n,i=1,\ldots ,N$, and *n* is the number of node in the attributed graph G. Then, the community detection problem transfers to an optimization problem that finds a consensus community structure $P^{*}$ from the candidate community partitions P. The main concern of WCMFA is the fusion strategy that could guarantee the final community partition $P^{*}$ is the optimum structure which would have different similarities with every partition $\{P_{i}|P_{i}\in P,i=1,\ldots ,N\},$ which is generated by the base community detection algorithm. In general, the consensus community partition $P^{*}$ should satisfy the following properties:

- $P^{*}$ should be robustness to small variations in P, according to the fusion strategy;
- $P^{*}$ should have better performance than each candidate community partition P∈P, statistically;
- $P^{*}$ should be similar to all single community partition.

The robustness property means that we assume the consensus partition $P^{*}$ are invariant to small perturbations in P, according to the community fusion strategy. In addition, well performance means that the partition $P^{*}$ is the optimum one in terms of ground truth.

### 3.2. Two-Layer Representation and Community Fusion

As is shown in the previous sections, we first introduce a two-layer hierarchical structure to represent attributed network data. Then, we introduce a weighted co-associate matrix based community fusion method taking both lower-layer and higher-layer representation into account. It is a significant difference between our approach and the existing community detection algorithms which employee the fusion strategy at the lower-layer representation.

**Definition** **1** (Base Community Detector). *Given an attributed network G(V,E,A), we define the base community detector $f=\{f^{t}\left(G\right),f^{a}\left(G\right)\}$ as the community detect algorithm that could apply to network topological structure data G(V,E), i.e.,*

$$f^{t}\left(G\right):G\to P,\;G\in G,$$

*where P is a community partition, and the clustering algorithm which could apply to node attributes A, i.e.,*

$$f^{a}\left(G\right):A\to P,\;A\in G.$$

Given node attributes A, we can obtain a community partition $P=\{C_{1},C_{2},\ldots ,C_{m}\}$ which satisfies $\cup_{i}C_{i}=V$ and $\forall C_{i},C_{j}\in P,C_{i}\cap C_{j}=\emptyset$, according to the base community detector $f^{a}$. Suppose the base community $C_{i}=\left\{v_{1},\ldots ,v_{l}\right\}\left(l\leq N,\forall v_{i}\in R^{d}\right)$ is a finite independent and identically distributed (IID) node set with attributes. A probability density estimate $\hat{p}\left(v\right)$ can be obtained from $C_{i}$ by using kernel density estimation (KDE), i.e.,

(7) $$\hat{p}\left(v\right)=\frac{1}{{\left(2\pi \right)}^{d/2}lh^{d}}\sum_{i=1}^{l}\exp\left(-\frac{1}{2h^{2}}{\left(v-v_{i}\right)}^{T}\left(v-v_{i}\right)\right),$$

where *h* is the window width. For each node $v_{k}\in C_{i}$, the class-conditional probability $p(v_{k}|C_{i})$ is

(8) $$p\left(v_{k}|C_{i}\right)=\frac{1}{{\left(2\pi \right)}^{d/2}lh^{d}}\sum_{i=1}^{l}\exp\left(-\frac{1}{2h^{2}}{\left(v_{k}-v_{i}\right)}^{T}\left(v_{k}-v_{i}\right)\right).$$

For simplicity, we use the mean of $C_{i}$, i.e., $\bar{v}$, to replace $v_{i}$; then, we have

(9) $$p\left(v_{k}|C_{i}\right)=\frac{1}{{\left(2\pi \right)}^{d/2}h^{d}}\exp\left(-\frac{1}{2h^{2}}{\left(v_{k}-\bar{v}\right)}^{T}\left(v_{k}-\bar{v}\right)\right).$$

If $v_{t}\in C_{i}$, then the co-occurrence probability of $v_{k},v_{t}$ in $C_{i}$ is

(10) $$\begin{array}{cc} p(v_{k},v_{t}|C_{i}) & =\frac{1}{{\left(2\pi \right)}^{d}h^{2d}}\exp\left(-\frac{1}{2h^{2}}\left[{\left(v_{k}-\bar{v}\right)}^{T}\left(v_{k}-\bar{v}\right)+{\left(v_{t}-\bar{v}\right)}^{T}\left(v_{t}-\bar{v}\right)\right]\right)\\ & =\frac{1}{{\left(2\pi \right)}^{d}h^{2d}}\exp\left(-\frac{1}{2h^{2}}\left[{\left(v_{k}-v_{t}\right)}^{T}\left(v_{k}-v_{t}\right)+2{\left(v_{k}-\bar{v}\right)}^{T}\left(v_{t}-\bar{v}\right)\right]\right). \end{array}$$

As $v_{k}$ and $v_{t}$ are independent and identically distributed, then $E[{\left(v_{k}-\bar{v}\right)}^{T}\left(v_{t}-\bar{v}\right)]=0$; then, we have

(11) $$\begin{array}{cc} p(v_{k},v_{t}|C_{i}) & =\frac{1}{{\left(2\pi \right)}^{d}h^{2d}}\exp\left(-\frac{1}{2h^{2}}{\left(v_{k}-v_{t}\right)}^{T}\left(v_{k}-v_{t}\right)\right)\\ & =\frac{1}{{\left(2\pi \right)}^{d}h^{2d}}\exp\left(-\frac{1}{2h^{2}}{∥v_{k}-v_{t}∥}^{2}\right), \end{array}$$

where ${∥v_{k}-v_{t}∥}^{2}$ denotes the Euclidean distance between $v_{k}$ and $v_{t}$. Now, we can see that the $p(v_{k},v_{t}|C_{i})$ is determined by $∥v_{k}-v_{t}∥$. In addition, with the window width *h* fixed, there exists a negative correlation between $p(v_{k},v_{t}|C_{i})$ and $∥v_{k}-v_{t}∥$. Therefore, we have the following definition.

**Definition** **2** (Related Attribute Similarity Matrix). *Suppose that a community partition $P=\{C_{1},C_{2},\ldots ,C_{m}\}$ is generated by the base community detector $f^{a}$ and node attributes A; then, the related attribute similarity of $v_{k},v_{t}$ in the same community $C_{i}\left(C_{i}\in P\right)$ is defined as*

(12) $$\psi \left(v_{k},v_{t}\right)=1-\frac{∥v_{k}-v_{t}∥}{\max_{v_{p},v_{q}\in C_{i}}∥v_{p}-v_{q}∥}.$$

*Note that the number of community $C_{i}$ which $v_{k},v_{t}$ belongs to is only one in P. Then, the matrix *Ψ* is defined as*

(13) $$\Psi_{kt}=\left\{\begin{array}{cc} \psi (v_{k},v_{t}), & if\;v_{k},v_{t}\;in\;the\;same\;community;\\ 0, & otherwise. \end{array}\right.$$

**Definition** **3** (Two-Layer Representation). *Given an attributed network G(V,E,A), we define the raw data G(V,E) and A as the lower-layer representation $R^{low}$ for the attributed network G, i.e.,*

(14) $$R^{low}=\left\{G\left(V,E\right),A\right\}.$$

*Given a set of base community detector, i.e., $F={\left\{f_{i}\right\}}_{i=1}^{N},i=1,2,\ldots N$, we apply it to the lower-layer representation, and it will generate some outputs, that is, the basic community partitions $\left\{P^{i}|P^{i}=f_{i}\left(G\right),i=1,2,\ldots ,N\right\}$ and the related attribute similarity matrix *Ψ*, which are defined as a part of higher-layer representation $R^{high}$, i.e.,*

(15) $$R^{high}=\langle {\left\{f_{i}\right\}}_{i=1}^{N},{\left\{P^{i}\right\}}_{i=1}^{N},{\left\{\Psi^{i}\right\}}_{i=1}^{N}\rangle ,\;P^{i}=f_{i}\left(G\right),f_{i}\in F,i=1,2,\ldots N.$$

*Note that, if $f_{i}$ is a base community detector for topological structure, then $\Psi^{i}=0$. Therefore, the two-layer representation of an attributed network G is $R=\{R^{low},R^{high}\}$.*

**Definition** **4** (Weighted Co-Association Matrix). *Given the two-layer representation $R=\{R^{low},R^{high}\}$ of the attributed network G and the partition weighting vector, $w=(w_{1},w_{2},\ldots ,w_{N})$, for each node pair accompanying with each partition $P^{k}$, the weighted co-association matrix C is defined as*

(16) $$C_{ij}=\frac{1}{N}\sum_{k}^{N}w_{k}\left(v_{i},v_{j}\right)\cdot \delta \left(P^{k}\left(v_{i}\right),P^{k}\left(v_{j}\right)\right),\;\forall v_{i},v_{j}\in V,P^{k}\in P$$

*and*

(17) $$w_{k}\left(v_{i},v_{j}\right):V\times V\to \left[0,1\right],$$

*where $P^{k}\left(v_{i}\right)$ represents the cluster label of node $v_{i}$ in the partition $P^{k}$, and δ(b,d) is 1, if b=d, and 0 otherwise. If we let w={1,…,1}, then the weighted co-association matrix becomes the co-association matrix, which is defined in Ref. [53]. For the network topological data G(V,E), the weighting function $w_{k}\left(v_{i},v_{j}\right)$ could be defined as the topological similarity such as Jaccard similarity coefficient, i.e.,*

(18) $$w_{k}\left(v_{i},v_{j}\right)=\left\{\begin{array}{cc} \frac{|\Gamma \left(v_{i}\right)\cap \Gamma \left(v_{j}\right)|}{|\Gamma \left(v_{i}\right)\cup \Gamma \left(v_{j}\right)|}, & \delta (P^{k}\left(v_{i}\right),P^{k}\left(v_{j}\right))>0,\\ 0, & otherwise, \end{array}\right.$$

*where $\Gamma (v_{i})$ is a function to measure the neighbors of node $v_{i}$ in G(V,E). Correspondingly, $w_{k}\left(v_{i},v_{j}\right)$ also could be defined as the related attribute similarity $\psi ({\tilde{v}}_{i},{\tilde{v}}_{j})$, i.e.,*

(19) $$w_{k}\left(v_{i},v_{j}\right)=\left\{\begin{array}{cc} 1-\frac{∥{\tilde{v}}_{i}-{\tilde{v}}_{j}∥}{\max_{{\tilde{v}}_{p},{\tilde{v}}_{q}\in C_{i}}∥{\tilde{v}}_{p}-{\tilde{v}}_{q}∥}, & \delta (P^{k}\left(v_{i}\right),P^{k}\left(v_{j}\right))>0,\\ 0, & otherwise, \end{array}\right.$$

*where ${\tilde{v}}_{i}$ represents the feature vector of node $v_{i}$ in the node attributes A, ∥·∥ is the norm operator. $C_{i}$ represents the cluster which has the label $P^{k}\left(v_{i}\right)$. If the $\delta (P^{k}\left(v_{i}\right),P^{k}\left(v_{j}\right))>0$ is satisfied, then we have $C_{i}=C_{j}$.*

Obviously, the matrix C could be viewed as a similarity matrix for the node set *V* in attributed network G. In addition, the more nodes $v_{i}$ and $v_{j}$ appear in the same communities, the more similar they are. Unlike the co-association matrix [53], we consider the similarity between any two nodes taking both lower-layer representation and higher-layer representation into account. The weighted co-association matrix is divided into two parts, i.e., the weighting part and the partition part. We first take a glance at the co-occurrence of two nodes in the same community partition, which is the data form higher-layer representation. If the node pair is indeed in the same partition, then we will calculate the degree of its similarity in detail by using lower-layer representation data, otherwise, ignore it. The core idea of weighted co-association matrix avoids the case where the node co-occurrence degree is only 0 and 1, and it is in line with the idea of human being to deal with the problem from the whole to the local. In other words, two-layer representation provides a Comprehensive perspective for attributed networks, while the other methods only consider the single view of attributed networks, which is prone to fall into the trap of local optimization.

### 3.3. Community Fusion Algorithm

Based on the weighted co-association matrix, then the community fusion algorithm, i.e., WCMFA, is constructed by using clustering algorithm, which could generate the consensus partition $P^{*}$. The following Algorithm 1 outlines our proposed community detection algorithm.

**Algorithm 1** The weighted co-association matrix based community fusion algorithm, WCMFA, which detects community structure in attributed networks based on the two-layer representation
**Input:** G=〈(G(V,E),A)〉: an attributed network; ${\left\{f\right\}}_{i=1}^{N}$: a set of base community detector; *N*: the number of candidate community partitions; *M*: the total number of node in the attributed network G; $sim^{t}\left(\cdot ,\cdot \right)$: the node pair similarity measure for topological structure; $sim^{a}\left(\cdot ,\cdot \right)$: the node pair similarity measure for attribute set; M(·): a similarity matrix based clustering algorithm.**Output:** $P^{*}$: the consensus community partition.1:$R^{low}\leftarrow G\langle G\left(V,E\right),A\rangle$;2://calculate the candidate community partitions P;3:
**for** i=1,2,…,N **do**
4:    **if** $f_{i}$ is an instance of $f^{t}$ for G(V,E) **then**5:        $P\left[i\right]\leftarrow f_{i}^{t}\left(G\right)$;6:    **else**7:        $P\left[i\right]\leftarrow f_{i}^{a}\left(G\right)$;8:        calculate the matrix $\Psi^{i}$ associated with P[i];9:    **end if**10:
**end for**
11:$R^{high}\leftarrow \langle {\left\{f_{i}\right\}}_{i=1}^{N},P,{\left\{\Psi^{i}\right\}}_{i=1}^{N}\rangle$;12://community fusion;13:
**for** i=1,2,…,N **do**
14:    **if** $P^{i}$ is generated by $f^{t}\left(G\right)$ **then**15:        $w_{i}\left(\cdot ,\cdot \right)\leftarrow sim^{t}\left(\cdot ,\cdot \right)$;16:    **else**17:        $w_{i}\left(\cdot ,\cdot \right)\leftarrow sim^{a}\left(\cdot ,\cdot \right)$;18:    **end if**19:
**end for**
20:
**for** u=1,2,…,n **do**
21:    **for** v=1,2,…,n **do**22:        **if** u≥v **then**23:           continue;24:        **end if**25:        $C_{uv}\leftarrow 0$;26:        **for** k=1,2,…,N **do**27:           $C_{uv}\leftarrow C_{uv}+w_{k}\left(u,v\right)\cdot \delta \left(P^{k}\left(u\right),P^{k}\left(v\right)\right)$;28:        **end for**29:        $C_{uv}\leftarrow \frac{1}{N}\cdot C_{uv}$;30:    **end for**31:
**end for**
32:$C\leftarrow C+C^{T}$;33:$P^{*}\leftarrow M\left(C\right)$;34:return the consensus community partition $P^{*}$.


In Algorithm 1, M(·) can be any kind of clustering algorithm which requires a similarity matrix as input, such as Single Link, Complete-Link, Average-Link, etc. [54].

## 4. Experiments

The empirical study of the WCMFA is given in this section. We first set up the experiments by introducing the datasets and comparison methods. Then, we evaluate the performance in terms of some criteria compared with other methods.

### 4.1. Experiment Setup

**Data sets**: in order to test the performance of our method, we selected three networks with node attributes: the counselor relationship network (Consult) [15], the London gang relationship network (London Gang) [55] and the Montreal gang relationship network (Montreal Gang) [56].

Consult is an attributed network that describes the relationship between employees in a consulting company. The topological structure of Consult is represented by a graph where a node is an employee and an edge is a relationship between two employees. In addition, every node has a feature set such as the organisation level (1: Research Assistant; 2: Junior Consultant; 3: Senior Consultant; 4: Managing Consultant; 5: partner), gender(1: Male; 2: Female), region (1: Europe; 2: USA), and location (1: Boston; 2: London; 3: Paris; 4: Rome; 5: Madrid; 6: Oslo; 7: Copenhangen), etc.

London gang: the attributed network is on co-offending in a London-based inner-city street gang, 2005–2009. In addition, the data comes from anonymised police arrest and conviction data for ’all confirmed’ members of the gang. The topological structure of London gang consists of 54 persons as nodes and the relationship between nodes as edges. In addition, every node also has a attribute set to describe the features of it such as Age, Birthplace, Residence, Arrests, Convictions, Prison and Music, etc.

Montreal gang: the data obtained form the Montreal Police’s central intelligence base, the Automated Criminal Intelligence Information System (ACIIS), was used to reconstruct the organization of drug-distribution operations in Montreal North. The topological structure of Montreal gang consists of 35 nodes with its interactions. Every node has a feature set such as Gang affiliation (1: Bloods; 2: Crips, 3: Other), Gang Ethnicity (1: Hispanic, 2: Afro-Canadian; 3: Caucasian; 4: Asian; 4: No main association/mixed) and Territory data (1: Downtown; 2: East; 3:West), etc.

**Comparison Methods and Base Community Detector Settings**: We select LPA [7] + CNS [15], denoted as LPACNS, BGLL + CNS [15], denoted as BGLLCNS, kMedoids + CNS [15], denoted as KmedCNS to perform the comparison experiment. These community detection methods incorporate node attribute similarities into edge weights by using the coupled similarity measure, i.e., CNS, and execute LPA, BGLL and kMedoids to detect community structure, respectively. As is discussed in the previous sections, we classified these methods as lower-layer fusion algorithms. In our proposed method, WCMFA, we select LPA, BGLL as the base community detector for network topological structure data and k-Means, k-Medoids as the base community detector for network attributes, in order to generate the higher-layer representation for attributed networks.

**Evaluation Metric**: For attributed networks with known community structure, we use the following criteria to evaluate the performance of WCMFA compared with other methods. Suppose $P=\{P_{1},P_{2},\ldots ,P_{K}\}$ is the result community partition generated by the selected method above, and $O=\{O_{1},O_{2},\ldots ,O_{L}\}$ is the ground-truth community structure. Then, we have the following evaluation metrics.

Rand Index (RI) [57]: Let *a* be the number of pairs of elements in *V* that are in the same subset in *P* and in the same subset in *O*, and *b* be the number of pairs of elements in *V* that are in different subsets in *P* and in different subsets in *O*, then we have

(20) RI=a+bn2.

Adjust Rand Index (ARI) [58]: Let $n_{kl}=\left|P_{k}\cap O_{l}\right|$, $b_{k}=\sum_{l=1}^{L}n_{kl}$ and $d_{l}=\sum_{k=1}^{K}n_{kl}$, then we have the following definition about ARI, i.e.,

(21) ARI=∑klnkl2−∑kbk2∑ldl2n212∑kbk2+∑ldl2−∑kbk2∑ldl2n2.

Normalized Mutual Information (NMI) [59]:

(22) $$NMI=-\frac{2\cdot \sum_{k=1}^{K}\sum_{l=1}^{L}\frac{|P_{k}\cap O_{l}|}{\left|V\right|}\log\left(\frac{\left|V|\cdot \right|P_{k}\cap O_{l}|}{|P_{k}\left|\cdot \right|O_{l}|}\right)}{\sum_{k=1}^{K}\frac{|P_{k}|}{\left|V\right|}\log\left(\frac{|P_{k}|}{\left|V\right|}\right)+\sum_{l=1}^{L}\frac{|O_{l}|}{\left|V\right|}\log\left(\frac{|O_{l}|}{\left|V\right|}\right)}.$$

For the attributed network without a known community structure, the modularity is a popular evaluate metric to test the performance of different methods. Weighted modularity [15] for attributed networks is defined as:

(23) $$WQ=\frac{1}{m^{w}}\sum_{i,j\in V}\left[A^{w}\left(i,j\right)-\frac{d_{i}^{w}d_{j}^{w}}{m^{w}}\right]\times \delta \left(c_{i},c_{j}\right),$$

where $A^{w}\left(i,j\right)$ is the weighted edge between node *i* and *j*, $d_{i}^{w}=\sum_{j}A^{w}\left(i,j\right)$, $m^{w}=\sum_{ij}A^{w}\left(i,j\right)$ and $c_{i}$ represents the community which the node *i* belong to. $\delta (c_{i},c_{j})$ is a delta function, that is, $\delta (c_{i},c_{j})=1$ if $c_{i}=c_{j}$; otherwise, it is 0.

### 4.2. Results

Table 1, Table 2 and Table 3 demonstrate the comparison results of the community detection algorithms: LPACNS, BGLLCNS, KmedCNS and WCMFA, which were performed on the attributed networks Consult, London Gang and Montreal Gang, respectively. Each column in all tables represents the corresponding results that all methods performed under one of evaluation metrics from RI, ARI, NMI and WQ. In addition, the last rows of three tables are the minimum performance difference between these methods on all evaluation metrics. Furthermore, numbers in bold style mean they are the best ones among all results in that column.

In terms of the Consult data, WCMFA is superior to LPACNS, BGLLCNS and KmedCNS in all evaluation metrics. For example, WCMFA gains 0.2261 improvement of ARI over the KmedCNS that is the best one among the other methods. Furthermore, WCMFA also achieves 0.0625 improvement of modularity over the best result 0.1345 which is generated by the KmedCNS. Similarly, WCMFA gains 0.113 and 0.1822 improvement of RI and NMI over the best results that are achieved by the other methods, respectively.

In terms of the London Gang data, WCMFA is superior to LPACNS, BGLLCNS and KmedCNS in all evaluation metrics, except the NMI. From Table 2, it is clear that WCMFA gains 0.0249, 0.0359 and 0.0455 improvement of RI, ARI and WQ over the best results that are obtained by the other methods, respectively. However, on the metric of NMI, WCMFA is not as good as BGLLCNS (the gap is 0.01) but is still superior to the other methods.

From Table 3, we could see that WCMFA is superior to LPACNS, BGLLCNS and KmedCNS in all evaluation metrics in terms of the Montreal Gang data. WCMFA gains 0.084, 0.1827, 0.1413 and 0.1884 improvement of RI, ARI, NMI and modularity over the best results that are achieved by the other methods, respectively. In other words, WCMFA is the best choice to detect the community structure among all comparison methods for the Consult, London Gang and Montreal Gang data, except the metric NMI on Montreal Gang.

From the results above, we could see that our community detction algorithm, WCMFA, is better in most cases than the other methods in most evaluation metrics. The main reason is that the fusion strategy of topological structure and nodes attributes at lower-layer will induce the loss of information in attributed networks. Another reason is that the two-layer representation is less sensitive to the change of raw data. Obviously, the lower-layer fusion strategy is vulnerable to a variation of raw data, that is, the little change in the raw data should induce a big influence for the final output of the community detection algorithm. Although the variation of lower-layer data would influence the higher-layer representation, we could reduce the influence at the lowest degree by using community ensemble learning, i.e., using community fusion strategy which considered both lower-layer and higher-layer representation of data. Therefore, our two-layer representation can avoid the problem that exists in the lower-layer fusion strategy. In our viewpoint, the higher-layer data is an abstract of lower-layer data, and it enhances the representation ability of the raw data. Based on the two-layer representation, our community detection approach, WCMFA, could achieve better results than the methods with the lower-layer fusion strategy, because the weighted co-association matrix based fusion strategy could mine more knowledge to improve the quality of detected community structure by leveraging both lower-layer and higher-layer data to the greatest extent.

In summary, the WCMFA which has the capability to capture the inherent information from the two-layer representation of attributed networks could obtain more intrinsic knowledge from data, so that it is more conducive to achieve a better performance in the data environments of multi-source heterogeneous.

### 4.3. Impact of Varying Size of Candidate Community Partitions and Nodes

As is discussed in the previous sections, the time-consuming part of WCMFA is sensitive to the size of nodes and the size of the candidate community partitions which are generated by the base community detectors. Roughly speaking, the WCMFA has two-stages, i.e., the base community partition generation stage and the community fusion stage. In the base community partition generation stage, the WCMFA could select anyone of the state-of-the-arts community detection algorithms to detect the candidate community partitions. However, our work focus on the fusion stage, that is, the performance of the WCMFA mainly depends on the size of candidate community partitions and nodes. Therefore, in this section, we randomly generate community partitions to test the performance of the WCMFA with varying size of |P| and *N*.

**Figure 2:**
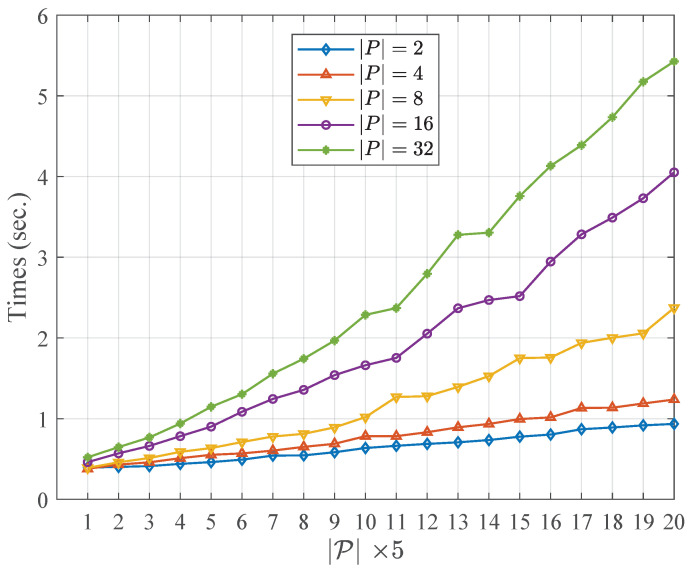
Comparison results with the varying size of |P|.

**Figure 3:**
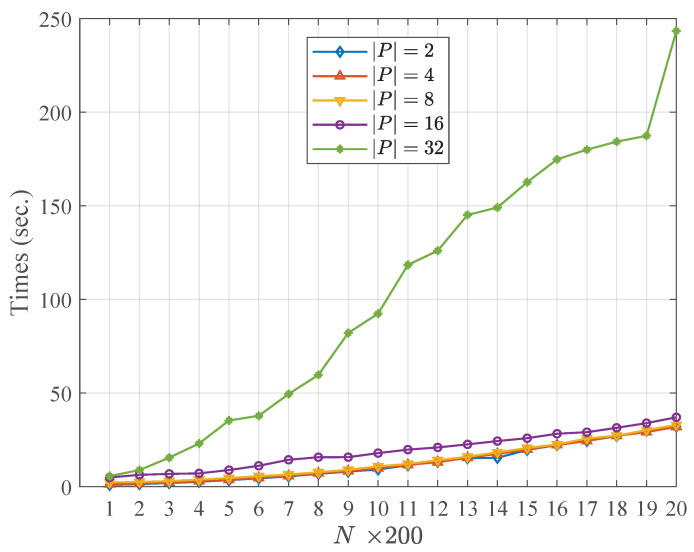
Comparison results with the varying size of *N*.

Table 4 and Table 5 describe the running time of the community fusion part of the WCMFA with the varying size of the candidate partitions and the nodes in networks, respectively. More specifically, the results shown in Table 4 and Table 5 are at the settings of n=200, |P|=2, respectively. Figure 2 and Figure 3 show the comparison results with the varying size of |P| and *N*, respectively. From the result above, we could see that the size of the candidate community partitions has approximately a linear correlation with the running time of the WCMFA. In other words, we can generate more candidate community partitions to improve the performance of the algorithm, and the time cost of the algorithm is not serious. However, the scale of the nodes in network plays an important role in the running time of the WCMFA. In Figure 3, the time curve rises sharply from 5.65 to 243.40 when the size of nodes ranges from 200 to 4000. In general, some parallel computing strategies that could be used to improve the bottleneck of algorithms which suffer the low efficiency of the large scale of data.

In summary, the WCMFA could generate more candidate community partitions to improve the performance with low time cost. Although the scale of data has more impacts than the size of |P|, it is useful to incorporate some parallel computing strategies to save the running time.

## 5. Conclusions

Due to the complexity of data and the lack of effective community fusion strategy, how to effectively and robustly detect the inherent community structure in the attributed networks is a challenging task. In this work, we developed a novel two-layer representation of data to capture the latent and inherent knowledge form attributed networks in a multi-source heterogeneous data environment, and proposed a multi-layer fusion strategy based community ensemble learning method, WCMFA, to detect the community structure from network data. It extends the community detection method from a single-view to a multi-view style, which is consistent with the thinking model of the human beings. Experiments show that our method is superior to the state-of-the-art community detection algorithms for attributed networks.

Several aspects of the new method are worth investigating in further depth, including how to select the number of layers, fusion strategies and the style of community ensembles, etc. In the future, we will focus on the hierarchical representation of attributed networks because a good representation will obtain an easy solution to solve the problem that seems complicated.

## Figures and Tables

**Table 1:** The comparison results with respect to the Consult.

Methods	RI	ARI	NMI	WQ
LPACNS	0.5855	0.1717	0.3182	0.1024
BGLLCNS	0.4889	0.0237	0.0014	0.0165
KmedCNS	0.8019	0.6038	0.6007	0.1345
WCMFA	**0.9150**	**0.8299**	**0.7828**	**0.1970**
Δ	0.1130	0.2261	0.1822	0.0625

**Table 2:** The comparison results with respect to the London Gang.

Methods	RI	ARI	NMI	WQ
LPACNS	0.3788	0.0015	0.0003	0.0000
BGLLCNS	0.5265	0.0138	**0.1108**	0.0032
KmedCNS	0.3753	0.0353	0.0359	0.0005
WCMFA	**0.5514**	**0.0712**	0.1008	**0.0487**
Δ	0.0249	0.0359	−0.0100	0.0455

**Table 3:** The comparison results with respect to the Montreal Gang.

Methods	RI	ARI	NMI	WQ
LPACNS	0.5513	0.2340	0.4312	0.0560
BGLLCNS	0.6639	0.0110	0.2064	0.0283
KmedCNS	0.7899	0.5146	0.6372	0.1151
WCMFA	**0.8739**	**0.6973**	**0.7785**	**0.3035**
Δ	0.0840	0.1827	0.1413	0.1884

**Table 4:** Running time with the varying size of candidate community partitions.

Candidate Partitions	The Number of Communities in One Partition |P|				
	2	4	8	16	32
5	0.39	0.38	0.39	0.46	0.52
10	0.40	0.43	0.46	0.57	0.65
15	0.41	0.46	0.51	0.66	0.77
20	0.44	0.51	0.59	0.78	0.94
25	0.46	0.55	0.64	0.90	1.15
30	0.49	0.57	0.71	1.09	1.30
35	0.54	0.60	0.78	1.24	1.56
40	0.55	0.65	0.81	1.36	1.74
45	0.58	0.69	0.89	1.54	1.97
50	0.64	0.78	1.02	1.66	2.29
55	0.66	0.78	1.27	1.75	2.37
60	0.69	0.83	1.28	2.05	2.79
65	0.71	0.89	1.39	2.37	3.28
70	0.74	0.93	1.53	2.47	3.30
75	0.78	1.00	1.75	2.52	3.76
80	0.80	1.02	1.76	2.95	4.13
85	0.87	1.13	1.94	3.28	4.39
90	0.89	1.14	2.00	3.49	4.73
95	0.92	1.19	2.06	3.73	5.17
100	0.94	1.24	2.37	4.05	5.43

**Table 5:** Running time with different node sizes.

Node Size	The Number of Communities in One Partition |P|				
	2	4	8	16	32
200	0.93	1.25	2.30	4.90	5.65
400	1.37	1.70	2.55	6.32	8.87
600	1.92	2.14	2.99	6.82	15.50
800	2.62	2.93	3.75	7.10	23.04
1000	3.50	3.67	4.62	8.86	35.34
1200	4.43	4.70	5.59	11.16	37.86
1400	5.51	5.74	6.49	14.37	49.48
1600	6.86	7.16	7.72	15.74	59.71
1800	8.08	8.35	9.00	15.76	82.09
2000	9.17	9.78	10.86	17.92	92.41
2200	11.47	11.66	12.18	19.80	118.44
2400	13.33	13.05	14.09	20.96	126.04
2600	15.26	15.42	16.01	22.60	145.14
2800	15.38	17.52	18.34	24.36	149.09
3000	19.68	19.84	20.66	25.90	162.64
3200	22.17	22.25	22.56	28.27	174.81
3400	24.45	24.42	25.76	29.11	179.99
3600	26.99	27.16	27.24	31.44	184.27
3800	29.83	29.14	30.22	33.93	187.42
4000	32.14	31.99	32.98	37.06	243.40
